# Smart Directional Liquid Manipulation on Curvature-Ratchet Surfaces

**DOI:** 10.1021/acsnano.4c18229

**Published:** 2025-01-30

**Authors:** Jiaqi Miao, Alan C. H. Tsang

**Affiliations:** Department of Mechanical Engineering, The University of Hong Kong, Hong Kong, China

**Keywords:** smart liquid manipulation, interfacial energy, heterogeneous surfaces, Laplace pressure, information encryption

## Abstract

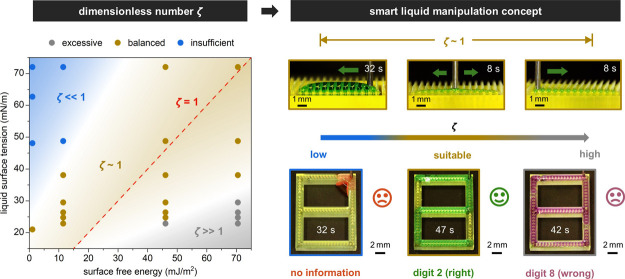

Structured surfaces leverage interfacial energy for directional liquid manipulation without external power, showing tremendous potential in microfluidics, green energy and biomedical applications. While the interplay of interfacial energy between solid surfaces and liquids is crucial for liquid manipulation, a systematic understanding of how the balance in liquid–solid interfacial energy affects liquid behaviors remains lacking. Here, using the curvature-ratchet surface as a generic example, we reveal the complex directional liquid dynamics inherent in the subtle regulation of liquid–solid interfacial energy. We show that curvature and tilt features regulate Laplace pressure asymmetry to enable directional, bidirectional and reverse liquid manipulation. These processes can be modulated by surface free energy and liquid surface tension, and we define their ratio as a new dimensionless number *ζ* to characterize the liquid–solid interfacial energy relationship. The balanced liquid control happens when *ζ* ∼ 1, which facilitates versatile liquid behaviors, e.g., fan-shaped spreading, gradient-induced redirection, and back-and-forth transport on various surface array arrangements, all resulting from matching structural designs with the proper *ζ*. Inspired by this, we showcase an innovative liquid-based information encryption technique, where the liquid displays correct information on preprogrammed surfaces only at designated *ζ* values. This study lays the groundwork for smart directional liquid manipulation and broadens its application domains.

## Introduction

Directional liquid manipulation,^1−5^ as a pivotal liquid management method, has shown great potential in various applications, e.g., energy harvesting,^6^ microfluidics,^7,8^ surface chemistry,^9,10^ and biomedical applications.^11,12^ Early studies have employed chemical gradient modifications^13^ or anisotropic wettability structures^14,15^ to enable liquid spreading with directionality. Yet, the magnitude of liquid movement is constrained by the range of chemical gradients or the inherent volumes/properties of liquids/droplets, making it hard to achieve stable liquid transport over a long distance. The solution to this dilemma is acquired from the unique liquid behaviors observed on natural surfaces, including the rapid liquid transport on the peristome surface of *Nepenthes alata* for capturing aquaplaning insects,^16^ the shorebirds’ repeatedly opening beaks for drinking,^17^ and the semiopen capillary channels on the Texas horned lizards’ skin surface for directing water.^18^ These natural examples bypass our imagination and provide valuable inspiration for engineered surface designs,^19−23^ which utilize unbalanced interfacial forces to guide liquid transport without energy input. Therefore, such bioinspired engineered surfaces can be easily coupled to various engineering systems, e.g., water collection devices,^24^ microfluidic channels,^8^ 3D printing equipment,^25^ and wearable sensor components.^26^

Liquid manipulation using structured surfaces is essentially based on the regulation of interfacial energy between solid surfaces and liquids. For instance, the rapid development of superwetting surfaces has shown rich functionalities,^1,3,27,28^ including superhydrophilic surfaces that efficiently capture liquids for water collection or self-transport/pumping of liquids,^29,30^ as well as superhydrophobic surfaces that strongly propel liquids to enable self-cleaning and lossless liquid manipulation.^31−33^ Beyond surface wettability, recent studies have uncovered various liquid behaviors influenced by differences in liquid surface tension.^34−38^ From a unified perspective, these results suggest that adjustments to the liquid–solid interfacial energy system, whether from the liquid or solid surface, can lead to changes in liquid behaviors. However, deeper research is still lacking in understanding how changes in interfacial energies affect liquid behaviors. This gap hinders our comprehension of achieving robust and versatile liquid control under a balanced liquid–solid interfacial energy system, as well as catalyzing new potential application domains.

In this work, we present curvature-ratchet surfaces that enable the modulation of liquid–solid interfacial energy to address how liquid behaviors can be regulated via balanced interfacial energy and illustrate the concept of smart directional liquid manipulation. We elucidate how curvature-ratchets exploit asymmetric Laplace pressure induced by structure asymmetry to regulate complex liquid dynamics, encompassing transition among unidirectional, bidirectional and reverse manipulation, all of which are closely linked to variations in the liquid–solid interfacial energy system. We introduce a new dimensionless number *ζ* to describe the liquid–solid interfacial energy relationship, defined as the ratio of surface free energy (*γ*_S_) to liquid surface tension (*γ*_L_), i.e., *ζ* = *γ*_S_/*γ*_L_. It turns out that *ζ* determines three liquid control regimes: excessive (*ζ* ≫ 1), balanced (*ζ* ∼ 1), and insufficient (*ζ* ≪ 1). Among these, the balanced regime supports a variety of adjustable liquid manipulation patterns, including fan-shaped spreading, gradient-induced redirection, and back-and-forth transport on different surface array arrangements. Notably, minor changes in *ζ* can trigger markedly different behaviors of liquids even on the same surface design. Hence, it is necessary to consider both liquid–solid interfacial energy relationships informed by *ζ* and structural designs to determine the ultimate liquid behaviors. Building upon this understanding, we present a novel liquid-based information encryption technique, where the injected liquid conveys correct information on programmed surface designs within a specific *ζ*, while deviations from this range result in no or incorrect information. Our results present a promising approach for smart directional liquid manipulation, which will have profound implications in engineering applications involving complex transitions in liquid behaviors.

## Results and Discussion

### Curvature-Ratchet Surfaces

**Figure 1:**
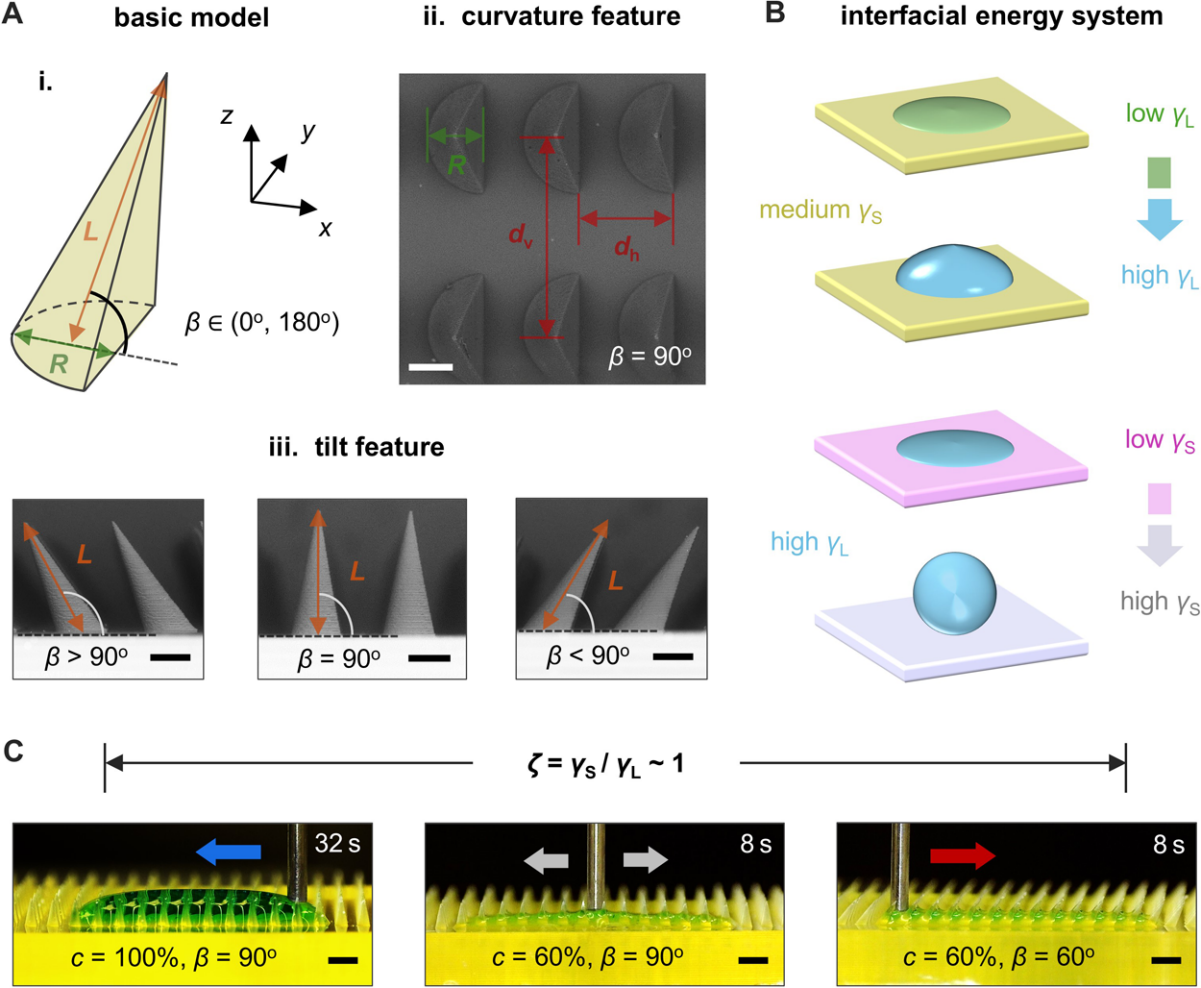
Overview of directional liquid manipulation on curvature-ratchet surfaces. (A) Illustration of curvature-ratchet surfaces: (i) basic model; and SEM images of (ii) curvature features and (iii) tilt features of various designs. (B) Varied liquid–solid interfacial energy system achieved by adjusting *γ*_L_ and *γ*_S_. (C) Directional, bidirectional and reverse liquid manipulation (left to right) within the balanced liquid control regime (*ζ* ∼ 1). Scale bars: 300 μm (A), and 1 mm (C).

We consider a curvature-ratchet model consisting of a semicircular bottom (radius *R* = 400 μm), a body length *L* = 1000 μm, and a structural angle *β* ranging from 0° to 180° (Figure 1A-i). High-precision structural features are ensured by the projection micro stereolithography technology (Figures S1 and S2). Figure 1A-ii,iii presents top and front-view scanning electron microscope (SEM) images (Experimental Section) of curvature-ratchets with standard array arrangements (horizontal spacing *d*_h_ = 700 μm; vertical spacing *d*_v_ = 1400 μm). These images show the uniform curvature structures and various tilt angle designs (*β* = 120°, 90°, and 60°). We emphasize the impact of varied liquid–solid interfacial energy systems on liquid dynamics (Figure 1B). The liquid takes on different forms according to the interplay between liquid surface tension and surface free energy, which in turn influences liquid transport behaviors in different regimes of *ζ*. In the identified regime for balanced liquid control (i.e., *ζ* ∼ 1, Figure 1C), curvature-ratchet surfaces can steadily guide directional, bidirectional, and reverse transport of various surface tension liquids (i.e., ethanol–water mixture), with mass fraction of DI water denoted by *c*, where a higher *c* refers to a higher surface tension (Figure S3).

### Directional Liquid Dynamics

We first investigate the directional liquid manipulation mechanism on curvature-ratchet surfaces to establish guidelines for on-demand design strategies of directional, bidirectional and reverse liquid control across 23–72 mN/m (*c* = 0–100%). We inject liquids on surfaces at 100 μL/min to create a surface-tension-dominant environment (Experimental Section). The liquid manipulation mechanism lies in regulating Laplace pressure (Δ*P*) at the 3D liquid–air interface, comprising two components: Δ*P*_c_ generated between curvature structures (top view) and Δ*P*_t_ produced between the ratchet and surface base (front view), where Δ*P* = Δ*P*_c_ + Δ*P*_t_. We define the Laplace pressure hindering liquid spreading as positive, and determine the transport direction by identifying the greater Δ*P* between the positive and negative directions of the *x*-axis (defined in Figure 1A-i).

**Figure 2:**
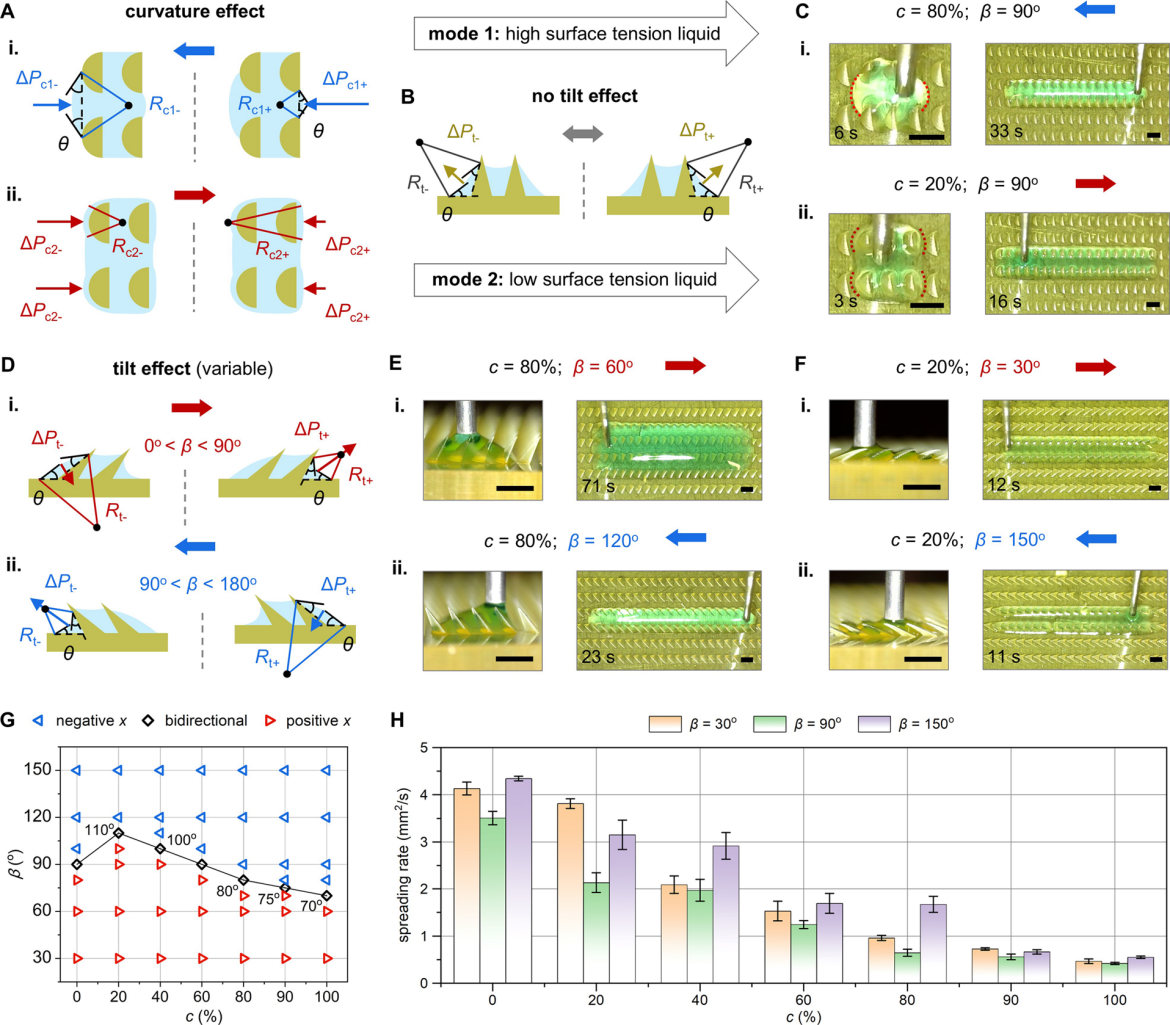
Directional liquid dynamics on curvature-ratchet surfaces (the arrow length/direction indicates the Laplace pressure magnitude/direction). (A) Curvature-mediated Laplace pressure asymmetry for (i) high surface tension liquids (mode 1) and (ii) low surface tension liquids (mode 2). (B) Symmetric Laplace pressure without tilt effects. (C) Directional spreading of (i) *c* = 80% liquid and (ii) *c* = 20% liquid. (D) Tilt-mediated asymmetric Laplace pressure: (i) *β* < 90°; (ii) *β* > 90°. (E) Directional spreading of *c* = 80% liquid on (i) *β* = 60° and (ii) *β* = 120° surfaces. (F) Directional spreading of *c* = 20% liquid on (i) *β* = 30° and (ii) *β* = 150° surfaces. (G) Phase diagram linking surface design to liquid spreading in different directions. (H) Spreading rates of various liquids on *β* = 30°, 90°, and 150° surfaces (mean ± SD). Scale bars: 1 mm.

We start by examining how the curvature feature influences Laplace pressure, with *β* set to 90° (i.e., the scenarios where no tilt feature is introduced). Like many other heterogeneous design strategies,^16,20^ the curvature feature disrupts the symmetry of liquid–air interfaces on structured surfaces,^4,23,37,38^ thereby effectively triggering the directional liquid manipulation. Intriguingly, traditional understanding suggests that a single asymmetric feature typically guides liquids in a fixed direction. However, we observe two liquid operation modes with opposite transport directions that switch with liquid surface tension (mode 1 and mode 2 in Figure 2A–C). For the high surface tension liquid (mode 1), the heterogeneity of ratchets, with one side being curved and the other side being flat, leads to distinct liquid–air interface characteristics at a stable contact angle *θ* (Figure 2A-i). The liquid curvature radius in the negative *x*-direction (*R*_c1–_) is greater than that in the positive *x*-direction (*R*_c1+_), resulting in a Laplace pressure gradient along the negative *x*-direction, described by
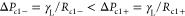
1where Δ*P*_c1–_ and Δ*P*_c1+_ refer to the Laplace pressure components between curvature structures along the negative and positive *x*-axis, respectively.

When *β* = 90°, the symmetric liquid–air interfaces on the sides of ratchets result in equal Laplace pressure components (Figure 2B), i.e., Δ*P*_t–_ = Δ*P*_t+_. Thus, the total Laplace pressures in the positive and negative *x*-directions, Δ*P*_1–_ and Δ*P*_1+_, are related as
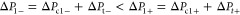
2

Following this mode, *c* = 80% liquid continuously breaks the pinning in the negative *x*-direction (Figure 2C-i), and achieves 10.4 mm directional transport distance within 33 s (Movie S1).

When the low surface tension liquid is injected (mode 2), it readily conforms to the ratchet profiles, creating distinct liquid–air interfaces on the curved side and the flat side (Figure 2A-ii). The liquid curvature radius in the negative *x*-direction (*R*_c2–_) becomes smaller than that in the positive *x*-directional (*R*_c2+_), leading to a larger Laplace pressure resistance along the negative *x*-direction (Δ*P*_c2–_ > Δ*P*_c2+_). Additionally, Δ*P*_t–_ and Δ*P*_t+_ remain equal, which results in a larger total Laplace pressure in the negative *x*-direction (Δ*P*_2–_ > Δ*P*_2+_). Figure 2C-ii illustrates this mode, where *c* = 20% liquid is directionally transported 12.7 mm in 16 s (Movie S1). This fast directional liquid transport mode is novel and occurs when liquids are extremely easy to spread across the surface due to low surface tension. However, in this case, the reduced resistance from heterogeneous structures makes liquid behaviors to be dominated by inertia, potentially leading to a shift from directional control to bidirectional spreading. As shown in Figure 2C-ii, the liquid slightly disrupts pinning in the negative *x*-direction before stabilizing in the positive *x*-direction.

We then consider the effects of the tilt feature (*β* ≠ 90°), where the Laplace pressure components Δ*P*_t–_ and Δ*P*_t+_ differ. For *β* < 90°, the heterogeneous liquid–air interfaces create less resistance in the positive *x*-direction, i.e., Δ*P*_t–_ > Δ*P*_t+_ (Figure 2D-i); for *β* > 90°, the relationship reverses and leads to Δ*P*_t–_ < Δ*P*_t+_ (Figure 2D-ii). As *β* deviates from 90°, the difference between Δ*P*_t–_ and Δ*P*_t+_ increases, which may surpass the Laplace pressure asymmetry from curvature, i.e., |Δ*P*_t+_ – *P*_t–_| > |Δ*P*_c+_ – *P*_c–_|. At this stage, the tilt feature directs the liquid spreading toward the ratchet’s inclined direction (Movie S1). For *c* = 80% liquid on *β* = 60° surfaces, the Laplace pressures due to the curvature and tilt features are competing against each other. Even if the tilt feature plays a dominant role in determining the resulting liquid transport in *x*-direction, the countering effect of the curvature feature causes the liquid to expand more rows along the *y*-axis and be transported only 13.4 mm within 71 s (Figure 2E-i). In contrast, on *β* = 120° surfaces, the synergistic effect of curvature and tilt features allows the liquid to quickly expand 13.8 mm in 23 s with fewer wetted rows along the *y*-axis (Figure 2E-ii). For *c* = 20% liquid on *β* = 30° surfaces (Figure 2F-i), curvature and tilt features jointly facilitate directional liquid transport along the positive *x*-direction, transporting 13.1 mm in 12 s. On *β* = 150° surfaces, the tilt feature dominates the spreading of *c* = 20% liquid toward the negative *x*-direction with a distance of 13.7 mm within 11 s (Figure 2F-ii).

Figure 2G presents a phase diagram linking surface design to liquid spreading toward negative *x*-direction (blue), positive *x*-direction (red), and bidirections (black). As *β* deviates from 90°, two curvature-mediated liquid manipulation modes shift to tilt-dominated spreading. We design ratchets with *β* = 70°, 80°, 100°, and 110° to identify the critical transition point for liquid transport directions. Notably, the reasons for bidirectional spreading of liquids at *c* = 0% and *c* = 60% on *β* = 90° surfaces differ: for *c* = 0%, the high wettability of the low surface tension liquid exceeds the controllable range of structural features; for *c* = 60%, it reflects an intermediate state between two opposing liquid transport modes. In Figure 2H, we measure the spreading rate of different liquids on three typical surface designs (*β* = 30°, 90°, and 150°). To account for the differences in liquid spreading along both the *x*-axis and *y*-axis, we use the liquid covered area per second to measure the spreading rate. We find that higher surface tension leads to increased resistance and reduced liquid spreading rate, and the same liquid exhibits faster transport on tilted surfaces due to enhanced Laplace pressure asymmetry. To comprehensively evaluate the directed liquid manipulation on curvature-ratchet surfaces, we investigate the spreading of extremely low/high surface tension liquids, and the impact of inertial effects on liquid transport (Figures S4 and S5). Especially, we illustrate that micro/nanoscale liquid–air interfaces fundamentally support the cross-scale applicability of curvature-ratchet structural features (Figures S6 and S7).

Overall, directional liquid manipulation relies on asymmetric interfacial forces from structural heterogeneity. In our ratchet model, the fixed curvature structure and varying tilt feature jointly contribute to the development of versatile liquid behaviors (Figure S8). Meanwhile, we emphasize that effective directional liquid steering is determined by the liquid–solid interfacial energy relationships, sensitivity to inertial effects, and cross-scale applicability. All these factors are crucial for effectively deploying structured surfaces in practical applications for robust and adjustable liquid manipulation.

### Liquid Control Regimes in the Varied Interfacial Energy System

**Figure 3:**
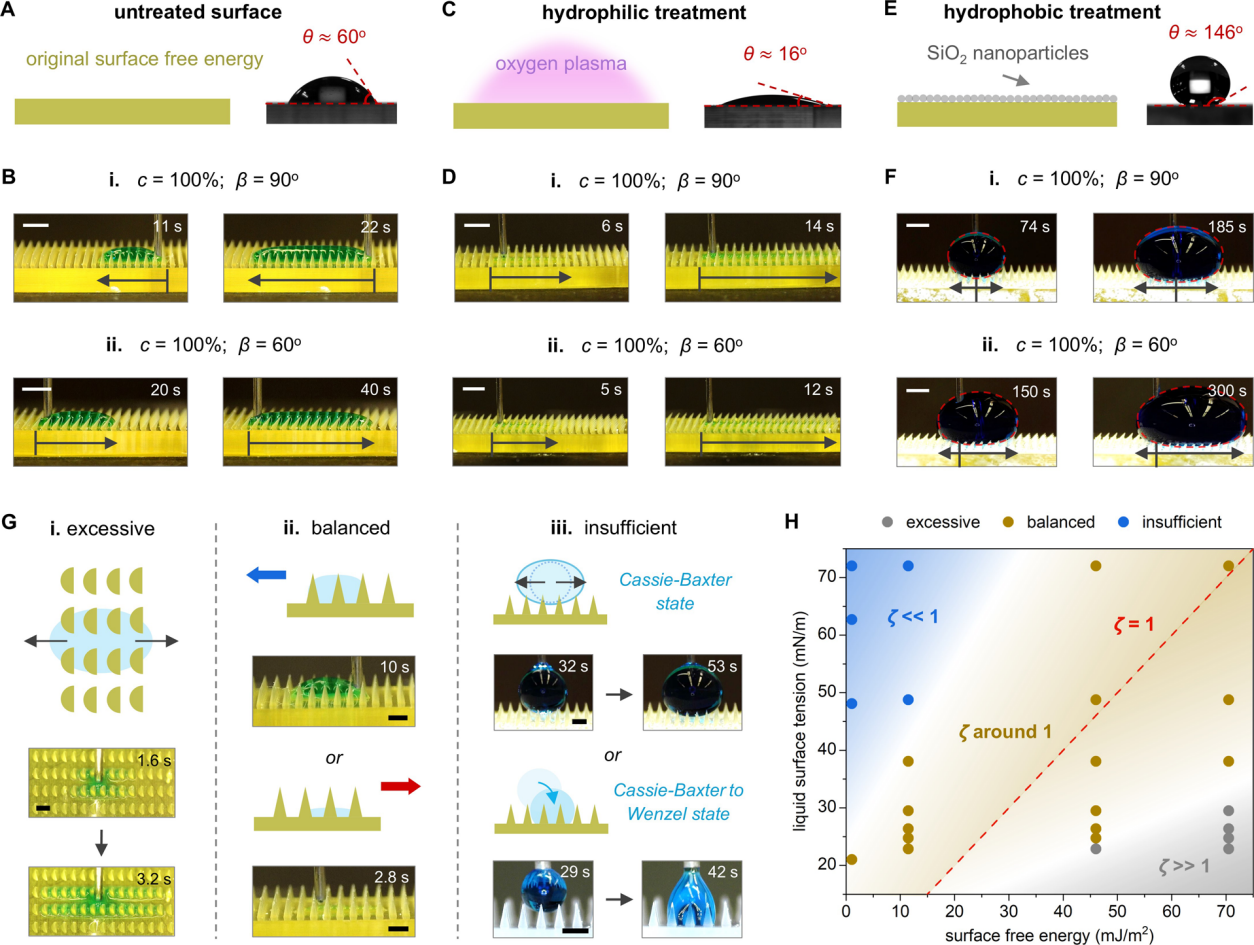
Liquid control regimes under varied liquid–solid interfacial energy systems. (A) Untreated surfaces with a ≈60° contact angle for *c* = 100% liquid. (B) Directional spreading of *c* = 100% liquid on untreated (i) 90° surfaces (11 and 22 s) and (ii) 60° surfaces (20 and 40 s). (C) Hydrophilic-treated surfaces with a ≈16° contact angle for *c* = 100% liquid. (D) Directional spreading of *c* = 100% liquid on hydrophilic-treated (i) 90° surfaces (6 and 14 s) and (ii) 60° surfaces (5 and 12 s). (E) Hydrophobic-treated surfaces with a ≈146° contact angle for *c* = 100% liquid. (F) Spreading of *c* = 100% liquid on hydrophobic-treated (i) 90° surfaces (74 and 185 s) and (ii) 60° surfaces (150 and 300 s). (G) Three liquid control regimes: (i) excessive (*c* = 0% liquid on untreated *β* = 90° surface, 1.6 and 3.2 s); (ii) balanced (*c* = 100% liquid on untreated *β* = 90° surface, 10 s; *c* = 20% liquid on untreated *β* = 90° surface, 2.8 s); (iii) insufficient (*c* = 100% liquid on hydrophobic-treated *β* = 90° surface, 32 and 53 s; *c* = 100% liquid on *β* = 90° Ecoflex 00–30 surface, 29 and 42 s). (H) Phase diagram of *ζ* and the various liquid control regimes. Scale bars: 2 mm (B, D, F); 1 mm (G).

The above investigation on directional liquid control via surface tension changes represents one effective method for tuning the liquid–solid interfacial energy system. Alternatively, here we investigate the tuning of the liquid–solid interfacial energy system by modifying surface free energy. In particular, we study the different liquid dynamics on hydrophilic/hydrophobic-treated surfaces (Movie S2). On untreated *β* = 90° and *β* = 60° surfaces, where the static contact angle of *c* = 100% liquid is ≈60° (Figure 3A), we show the reverse transport of *c* = 100% liquid as control experiments (Figure 3B-i,ii). After air plasma treatment (Experimental Section), the surface free energy increases and causes a static contact angle of ≈16° for *c* = 100% liquid (Figure 3C). On such surfaces, *c* = 100% liquid can be directed to the positive *x*-direction by *β* = 90° surfaces, displaying mode-2 spreading (Figure 3D-i). Upon introducing the tilt feature (*β* = 60°), the liquid spreads toward the positive *x*-direction (Figure 3D-ii). Therefore, with varied surface free energy, mode-2 spreading can also occur in high surface tension liquids, indicating that liquid behaviors are linked to the liquid–solid energy system rather than only liquid surface tension. Furthermore, by applying a superhydrophobic spray (Experimental Section), the reduced surface free energy leads to a static contact angle of ≈146° for *c* = 100% liquid (Figure 3E). The injected liquid, possessing relatively higher energy, repels the surface and exhibits the Cassie–Baxter state.^39^ In this case, the liquid is no longer influenced by the bottom curvature, presenting bidirectional spreading on *β* = 90° surface (Figure 3F-i). After introducing the tilt feature (*β* = 60°), the liquid spreads toward the positive *x*-direction (Figure 3F-ii), due to contact angle hysteresis.^40^

Based on the observed liquid behaviors, we categorize the liquid control regimes into excessive, balanced and insufficient to describe variations in the liquid–solid interfacial energy system. In the excessive control regime, high surface free energy minimizes the Laplace pressure resistance, and allows the liquid to easily overcome interfacial pinning and spread freely, resulting in the bidirectional spreading of *c* = 0% liquid on untreated *β* = 90° surface (Figure 3G-i). The balanced control regime describes an optimal energy relationship, which enables on-demand directional liquid manipulation (Figure 3G-ii). The insufficient control regime characterizes the low surface free energy that struggles to exert control over liquids (Figure 3G-iii). It includes (1) the full Cassie–Baxter state and (2) transitions to the Wenzel state^41^ under gravity. This regime is unsuitable for steady liquid manipulation, as it is highly susceptible to disturbances and prone to detachment from the surface. We characterize these regimes by the dimensionless number *ζ*. We examine the spreading of various surface tension liquids on untreated, hydrophilic/hydrophobic-treated, and soft material surfaces (Experimental Section). As illustrated in Figure 3H, *ζ* ∼ 1 corresponds to the balanced control regime; *ζ* approaching 0 signifies an insufficient control regime; while *ζ* significantly greater than 1 indicates the excessive control regime (see the detailed sample information on surfaces and liquids in Figure S9). This phase diagram suggests that steady liquid manipulation occurs when the energy systems of the liquid and solid surface are comparable. Additionally, critical transition values for different regimes may vary with surface design. For example, *c* = 0% liquid on an untreated *β* = 90° surface falls in the excessive control regime, but introducing tilt features shifts it to the balanced control regime (Figure 2G). The above regime distinction allows us to explore multimodal manipulation possibilities hidden in the robust balanced control regime, thus advancing smart liquid manipulation.

### Enriched Liquid Manipulation Patterns

Preprogrammed surface designs with specialized array arrangements can enrich liquid manipulation patterns compared to standard layouts (Figure 1A). This approach can introduce novel liquid manipulation modes and logical transport to enhance intelligence in liquid control.^35,42−46^ However, learning from the above results, the diversity of liquid behaviors also lies in the subtle regulation of the liquid–solid interface energy system, and this complex transformation has not been captured in previous studies. Here we conduct experiments in the balanced control regime (*ζ* ∼ 1) to explore how variations in the *ζ* value will affect liquid behaviors across identical surface designs with special array arrangements (Movie S3).

**Figure 4:**
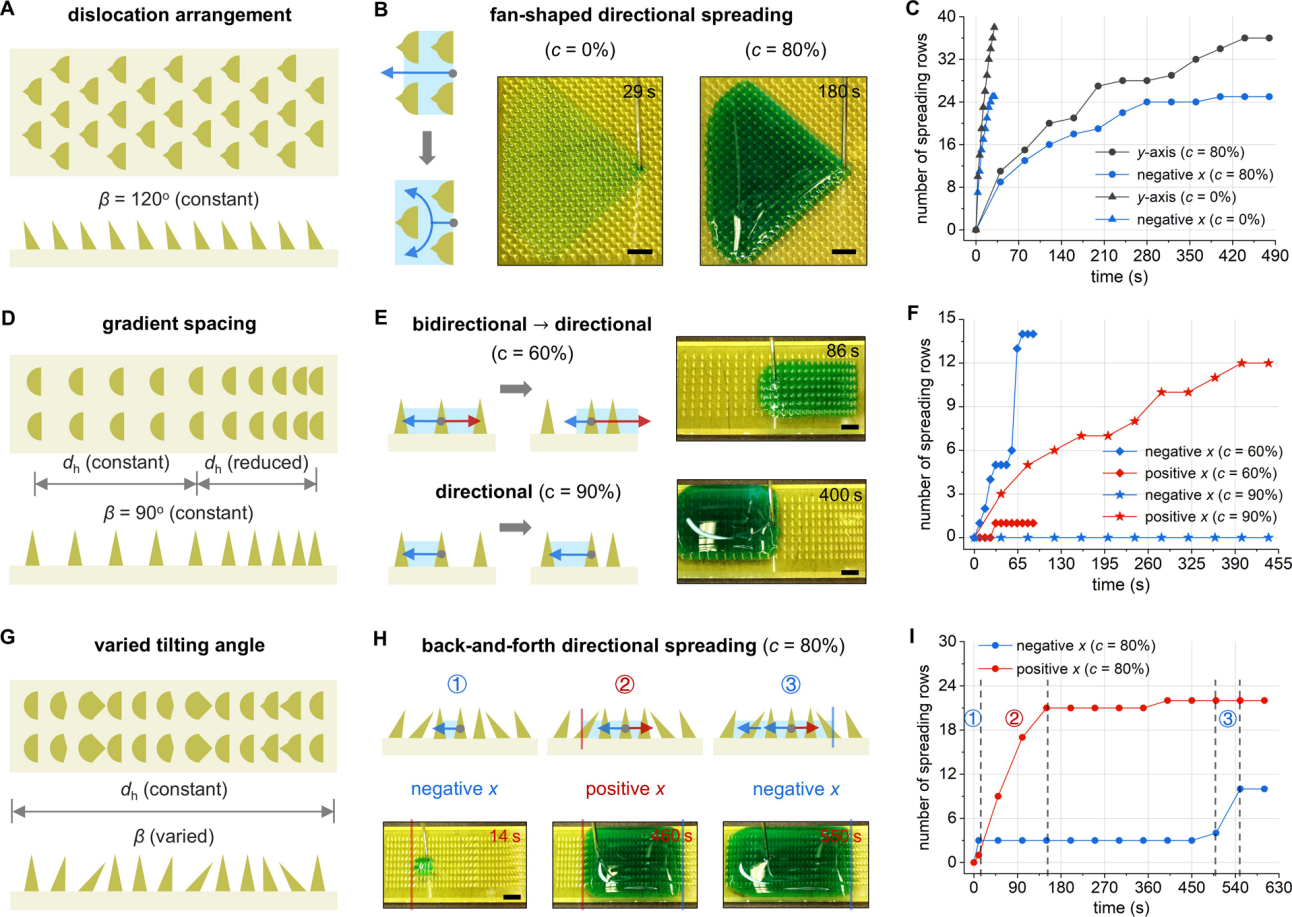
Enriched liquid manipulation patterns on diverse array arrangements. (A) Dislocation arrangement of curvature-ratchets. (B) Fan-shaped directional spreading of *c* = 0% (29 s) and *c* = 80% (180 s) liquids. (C) Number of spreading rows along the *y*-axis and the negative *x*-direction over time on the dislocation arrangement. (D) Gradient spacing arrangement of curvature-ratchets. (E) Directional spreading of *c* = 60% (86 s) and *c* = 90% (400 s) liquids. (F) Number of spreading rows along the positive and negative *x*-directions over time on the gradient spacing arrangement. (G) Varied tilting angle arrangement of curvature-ratchets. (H) Back-and-forth directional spreading of *c* = 80% liquid: stages ①–③ (14, 460, and 550 s). (I) Number of spreading rows along the positive and negative *x*-directions over time on the varied tilting angle arrangement. Scale bars: 3 mm.

We investigate the variation in liquid behaviors in three surface designs with different array arrangements (dislocation, gradient spacing, and varied tilting angle). Figure 4A shows curvature-ratchets with dislocation arrangements, all maintaining *β* = 120° (Figure S10). Rather than remaining confined within the gaps of two rows, the staggered ratchets compel the liquid to bifurcate, and ultimately result in a fan-shaped directional spreading (Figure 4B). Liquids at *c* = 0% and *c* = 80% have different liquid–solid interfacial energy relationships (*ζ* = 2.0 and *ζ* = 1.2); however, *β* = 120° curvature-ratchet surfaces ensure consistent transport directionality for both cases. The primary difference lies in the temporal aspect: the high wettability of *c* = 0% liquid facilitates a faster transport rate (Figure 4C).

Figure 4D illustrates an array arrangement with gradient spacing, where one-half has evenly spaced ratchets and the other half features decreasing *d*_h_, all with a consistent design angle of *β* = 90° (Figure S11). The surface with a denser ratchet arrangement provides greater guiding forces, allowing *c* = 60% liquid to shift from bidirectional to positive *x*-direction spreading (Figure 4E). By contrast, this design does not change the transport direction of *c* = 90% liquid, which originally moves in the negative *x*-direction (Figure 4F). Minor changes in liquid–solid interfacial energy, with *ζ* shifting from 1.56 to 0.94, trigger an opposite liquid transport direction. Both scenarios fall in the balanced control regime, the varied liquid behaviors induced by adjustments to the liquid–solid interfacial energy system enable on-demand tuning of liquid behaviors.

Figure 4G shows a ratchet array with evenly spaced but varying *β* (Figure S12). The different structural angles can locally alter the asymmetric Laplace pressure, guiding the liquid’s back-and-forth spreading (Figure 4H). *c* = 80% liquid initially spreads along the negative *x*-direction on *β* = 90° ratchets (stage ①); when it encounters backward-tilted ratchets (*β* < 90°), the interfacial resistance increases and forces the liquid to change direction toward the positive *x*-direction (stage ②); upon again meeting backward-tilted ratchets (*β* > 90°), the curvature effect facilitates the liquid’s escape from strong negative *x*-direction pinning (stage ③), allowing it to spread back along the negative *x*-direction after prolonged confinement from 150 to 450 s (Figure 4I). However, when using the higher surface tension *c* = 100% liquid (with *ζ* shifting from 1.2 to 0.64), such arrangement does not change the liquid’s directionality (Figure S12).

These results indicate that even with identical surface designs, subtle changes in the liquid–solid interfacial energy are vital in influencing the resulting behavior of liquids. Therefore, it is essential to consider the tuning of liquid–solid interfacial energy alongside surface designs to enhance smart directional liquid manipulation and broaden its application domains.

### Liquid-Based Information Encryption Technique

**Figure 5:**
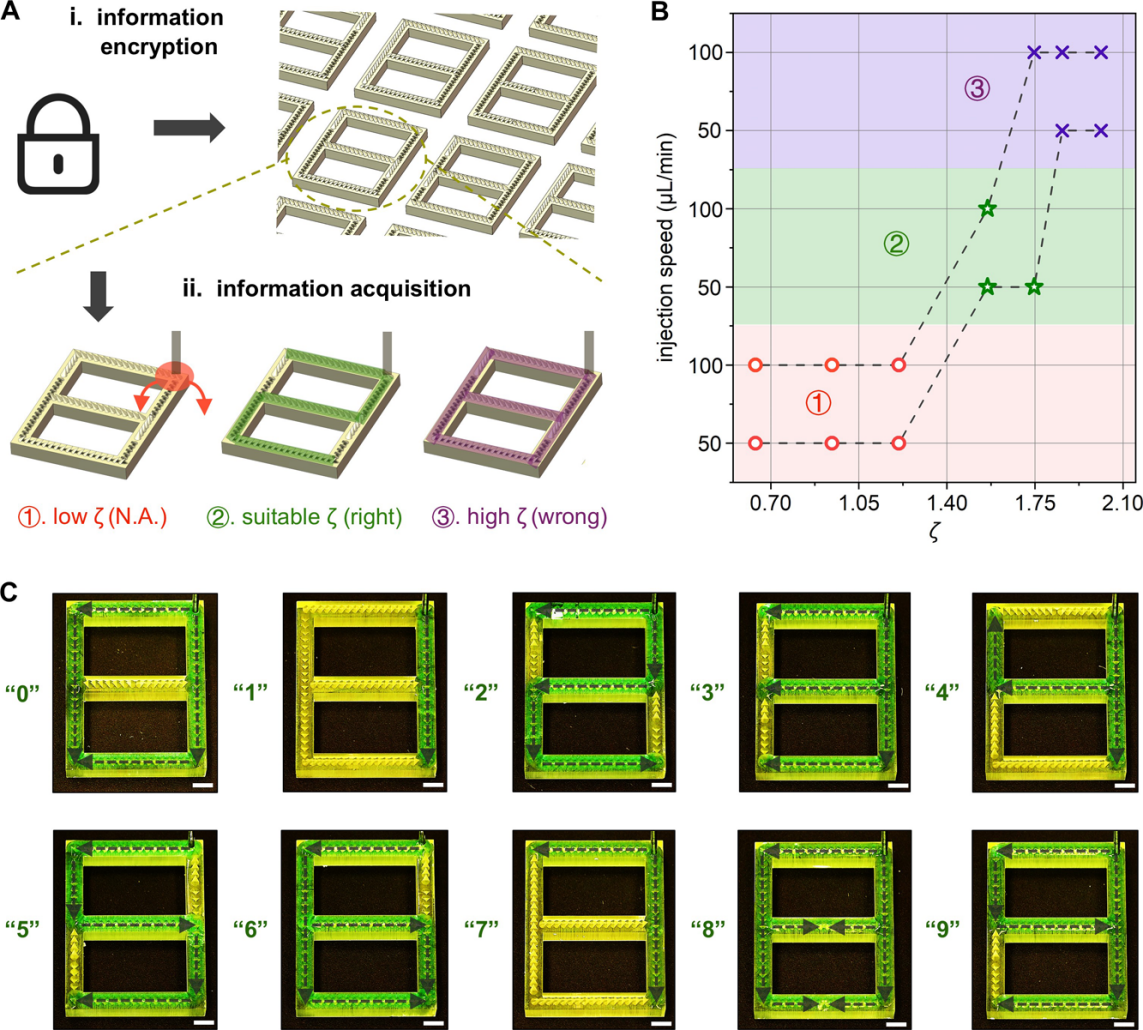
Liquid-based information encryption technique. (A) (i) information encryption; and (ii) three scenarios of information acquisition: ① no information, ② right information, and ③ wrong information. (B) Suitable *ζ* value ranges for obtaining the right information at various liquid injection speeds. (C) Experimental snapshots of *c* = 50% liquid used to decode digits 0–9 at the appropriate *ζ* values (gray dashed line arrows: indicating liquid transport trajectories). Scale bars: 2 mm.

Finally, we leverage the strong coupling of liquid–solid interfacial energy with surface designs to achieve smart liquid directional manipulation. We introduce a liquid-based information encryption technique that encodes information into preprogrammed surface designs (Figure 5A-i), where only specific *ζ* values serve as keys to access the right information (Figure 5A-ii). Specifically, low *ζ* values correspond to the insufficient liquid control regime with liquid accumulated close to the injection region, which eventually falls off the surface and reveals no information. Conversely, high *ζ* values lead to uncontrolled bidirectional transport, presenting wrong information. Only within an appropriate *ζ* range will the injected liquid convey the right information (Figure S13 and Movie S4). The implementation of this information encryption technique fundamentally relies on the combination of surface designs and appropriate liquid–solid interfacial energy systems, which aligns with the key points of our smart liquid manipulation concept.

Moreover, high-speed liquid injection tends to break the bidirectional pinning, entering the excessive control regime (Figure S5). Experimental tests in Figure 5B show that the 100 μL/min injection flow rate exhibits a narrower suitable range of *ζ* values compared to 50 μL/min. When the appropriate *ζ* value is chosen, the information embedded in the surface design becomes accessible upon liquid injection. Injecting *c* = 50% liquid with a flow rate of 50 μL/min at the same position, information-encoded surfaces can present different numbers, ranging from 0 to 9 (Figure 5C and Movie S4). Similar to the design concept with varied *β* in Figure 4G, strategically placing anchors at critical turning positions (see detailed array arrangements of ten digits in Figure S14) allows the liquid to display versatile types of information, including but not limited to numbers, letters, and shapes.

This liquid-based information encryption technique demonstrates an intriguing application of coupling the liquid–solid interfacial energy system and surface designs to regulate the presence or absence of information. This approach opens new opportunities for utilizing directional liquid manipulation in encoded information display.

## Conclusions

To conclude, we propose the concept of smart liquid manipulation via the regulation of liquid–solid interfacial energy on curvature-ratchet surfaces. Using the dimensionless number *ζ*, we define three liquid control regimes associated with changes in the liquid–solid interfacial energy system, where a balanced control regime occurs when *ζ* ∼ 1. In this regime, minor variations in the liquid–solid interfacial energy system can lead to different liquid behaviors, as evidenced by mode-1 and mode-2 liquid manipulation, as well as various liquid manipulation patterns across different array arrangements. Proper integration of *ζ* with surface designs is key to controlling directional liquid manipulation. Upon this understanding, we present an information encryption technique that utilizes smart liquid directional manipulation. The revealed intelligence is particularly relevant to microscale liquid management technologies and their diverse applications.

In this work, we capture diverse liquid dynamics under the same surface designs due to subtle changes of liquid–solid interfacial energy system. This discovery may address long-standing criticisms regarding the monotonous liquid behavior on fixed surfaces. Moreover, the identified balanced control regime at *ζ* ∼ 1 will be suitable for cases where using 3D surface morphology to guide liquid manipulation, exemplified by the curvature-ratchet surfaces discussed in this study. However, when shifting to guiding 2D liquid manipulation on superhydrophilic surfaces with hierarchical structures,^16,19^*ζ* ≫ 1 may fall in the suitable control regime. Conversely, for manipulating droplet bouncing on heterogeneous structured surfaces,^33,47,48^*ζ* ≪ 1 will be the suitable control regime, to ensure exceptionally low surface free energy and prevent liquid capture that hinders the expected bouncing behavior. Further, when considering surfaces with anomalous wetting properties, e.g., being both hydrophilic and oleophobic,^49,50^ only considering *ζ* as the criterion seems insufficient. These complexities call for future research to find suitable control regimes in different systems, which will lead to a unified framework for smart liquid manipulation. Undoubtedly, the proposed smart liquid manipulation concept will allow simple structured surfaces to exhibit complex dynamic liquid control abilities achievable only through sophisticated external energy intervention methods in the past, thereby enhancing their potential as viable candidates for addressing the challenges of future real-world applications.

## Experimental Section

### Materials

Ethanol (AR, water ≤0.3%), isopropyl alcohol (ACS, ≥99.5%), and potassium hydroxide (ACS) were provided by Aladdin Industrial Co., Ltd. (Shanghai, China). Ecoflex 00-30 was purchased from Smooth-on Inc. (Macungie, PA, USA). The superhydrophobic spray was purchased from Weijing Nanomaterials Co., Ltd. (Dongguan, China). Methylene blue (AR), safranin dye (2.5%), and crystal violet dye (2.5%) were acquired from BKMAM Biotechnology Co., Ltd. (Changde, China). Deionized water (DI water) was produced by the Direct-Q deionized water system (Millipore, MA, USA).

### Fabrication of Curvature-Ratchet Surfaces

The structured surfaces were fabricated using projection microstereolithography (BMF MicroArch S240; printing resolution: 10 μm). This high-precision 3D printing technology ensured the creation of various complex structures and fine dimensions for this study. After printing, the surfaces were sonicated in isopropyl alcohol for 15 min to remove uncured resin and then dried in an oven at 70 °C for 30 min to eliminate isopropyl alcohol. In Figure 3G-iii,H, we introduced soft material (Ecoflex 00–30) surfaces, obtained through a molding process. The process involved designing a mold based on the desired surface structure, treating it with air plasma (with a plasma cleaner from Harrick Plasma Inc.) for 15 min to remove impurities, pouring in the Ecoflex precursor (Ecoflex A and Ecoflex B in a mass ratio of 1:1), degassing in a vacuum for 10 min, and finally curing at room temperature (25 °C) for 8 h before demolding to acquire the surface.

### Microscopy

The SEM images of curvature-ratchets were observed using the Hitachi S-3400N scanning electron microscope.

### Characterization of Experimental Liquids

Surface tension and apparent contact angles for the experimental liquids (ethanol–water mixture) were measured using an optical video-based contact angle meter (Model 100SB, Sindatek Instrument Co., Ltd., New Taipei City, Taiwan). As the DI water content (*c*) increases, the liquid surface tension rises (Figure S3A). The apparent contact angle on the untreated surface material is depicted in Figure S3B, showing a positive correlation with liquid surface tension. To enhance visualization, the experimental liquids were dyed with methylene blue (0.2 mg/mL), which was confirmed to have no significant effect on the liquid surface tension (Figure S3C). Surface free energy was derived using the accompanying MagicDroplet software from the contact angle meter, employing the Fowkes method.

### Experimental Setup

This study utilized a liquid-infused model comprising several components: a syringe pump (LSP02-2B, Longer Pump), a 10 mL syringe (inner diameter: 14.8 mm) connected to soft tubes and a syringe needle (inner diameter: 0.3 mm), a 3-DOF *xyz* precision positioning platform (range: ± 6.5 mm along the *x* and *y* axes, 10 mm along the *z* axis; resolution: 0.03 mm), a hand-held microscope (Dino-Lite Edge AM4115ZTL, AnMo, Taiwan) paired with Dino Capture 2.0 software, a spirit level, and the surfaces. The liquid injection flow rate was typically set at 100 μL/min unless stated otherwise. The 3-DOF positioning platform allowed precise adjustment of the distance between the injection needle and the surface. Data from all experiments were processed using ImageJ software. The Weber number (*We*) was used to quantitatively confirm that the inertial effects could be neglected during directional liquid spreading, defined as
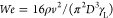
3where *ρ*, *v*, and *D* are the liquid density, injection flow rate, and the inner diameter of the syringe needle, respectively. With an injection flow rate at 100 μL/min, *We* is approximately 0.001 (Figure S5A), indicating that the inertial effect can be neglected. When aiming to consider the inertial effect, the required injection flow rates for achieving *We* = 1 and *We* = 5 could be determined using eq 3 (Figure S5B).

### Hydrophilic/Hydrophobic Surface Treatment

The hydrophilic treatment was achieved by exposing the surface to air plasma for 20 min. However, it was notable that this hydrophilic treatment is temporary, as the surface free energy will return to its original state after dozens of hours. The hydrophobic treatment involved thoroughly spraying the surface with a hydrophobic spray, followed by placing it in an 80 °C oven for 30 min. This process was repeated three times to ensure complete and uniform hydrophobization.

## Supporting Information Available

Directional liquid manipulation on curvature-ratchet surfaces (MP4)
Liquid spreading on untreated, and hydrophilic/hydrophobic-treated surfaces (MP4)
Multiple liquid manipulation patterns on different surface array arrangements (MP4)
Liquid-based information encryption technique (MP4)
3D printing setup for surface fabrication; characterization of liquid properties; comprehensive evaluations of liquid manipulation; design of various surface array arrangements; three information acquisition scenarios in the liquid-based information encryption technique; and detailed array arrangements for surfaces encoding ten-digit information (PDF)
