# Supplementary material for: Smart Directional Liquid Manipulation on Curvature-Ratchet Surfaces
Source: ACS Nano. 2025 Jan 30;19(5):5829–38. doi: 10.1021/acsnano.4c18229 (PMC11823629; doi:10.1021/acsnano.4c18229)
Supplement: Supplementary file 5 — nn4c18229_si_005.pdf [file nn4c18229_si_005.pdf]

Supporting Information for
"Smart Directional Liquid Manipulation on Curvature-Ratchet Surfaces"

Jiaqi Miao[†], Alan C. H. Tsang[†*]

This PDF file includes:

I. 3D Printing Setup and Surface Fabrication

II. Characterization of Liquid Properties

III. Mechanism Analysis of Liquid Manipulation

IV. Three Liquid Control Regimes

V. Design of Various Surface Array Arrangements

VI. Liquid-Based Information Encryption Technique

Figures S1-S14

Legends for Movies S1-S4

[*] [†] Department of Mechanical Engineering, The University of Hong Kong, Hong Kong, China. alancht@hku.hk

I. 3D PRINTING SETUP AND SURFACE FABRICATION

Figure S1A-i illustrates the components of the 3D printing system, including the CCD, beam splitter, projection lens, DMD, UV LED, membrane, resin, printing platform, and printing surfaces. A photograph of the printing system is shown in Figure S1A-ii. Taking $\beta = 60^o$ surface as an example, we illustrate the bottom-up layer-by-layer printing process (Figure S1B). The surface substrate was set to a maximum layer thickness of 40 µm to accelerate the printing speed (i.e., slice ①), while the curvature-ratchets were set to the smallest layer thickness of 10 µm to ensure precision (i.e., slices ② and ③).

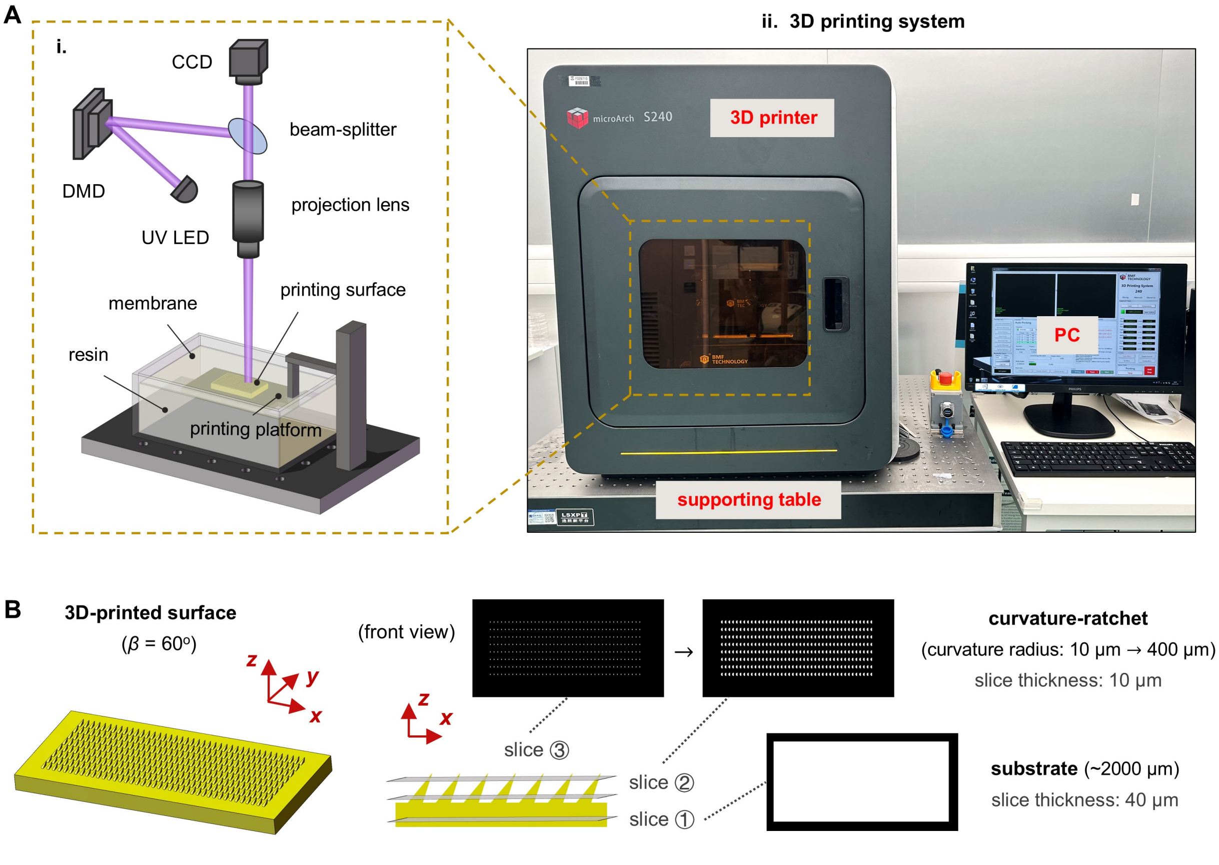

FIG. S1. (A) Illustration of i) the 3D printing components; ii) the 3D printing system. (B) The layer-by-layer printing of $\beta = 60^o$ surface.

Figure S2A-F presents the measured size parameters (R, L, d_h, d_v), and β of different surfaces, and compares them with the design values. The actual size measurements closely align with the design values.

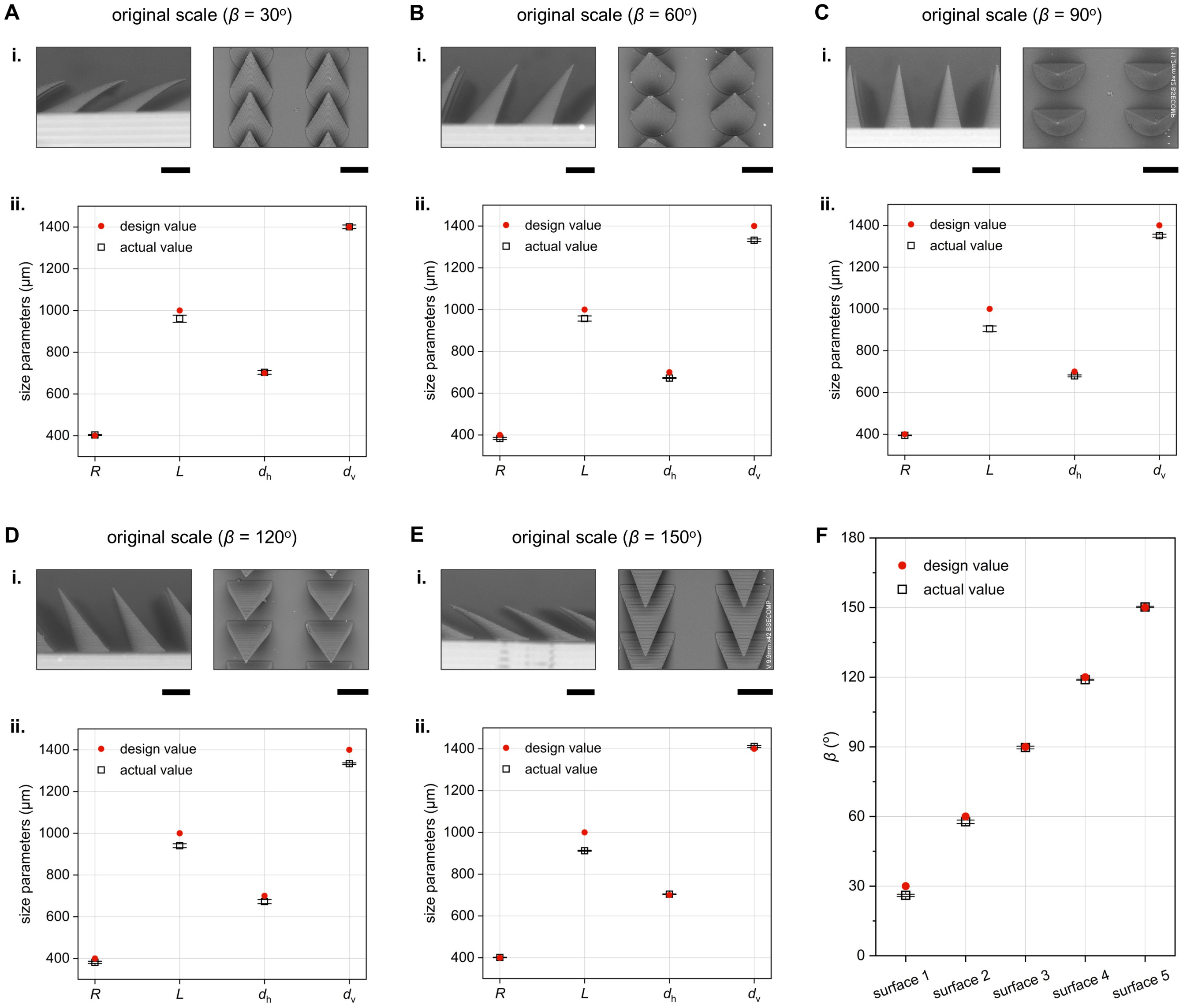

FIG. S2. (A) $\beta = 30^\mathrm{o}$ surface. (B) $\beta = 60^\mathrm{o}$ surface. (C) $\beta = 90^\mathrm{o}$ surface. (D) $\beta = 120^\mathrm{o}$ surface. (E) $\beta = 150^\mathrm{o}$ surface. (F) Comparison of the design value of β and its actual value. Error bars denote the SD. Scale bars: 300 μm.

II. CHARACTERIZATION OF LIQUID PROPERTIES

Figure S3A-B presents the typical interfacial properties of the experimental liquids, including surface tension and apparent contact angle (on untreated surfaces), as functions of DI water concentration c. Both the two properties increase with c. Notably, taking c = 90% liquid as an example, the introduction of methylene blue dye in our experiments does not significantly affect the liquid's interfacial properties (Figure S3C).

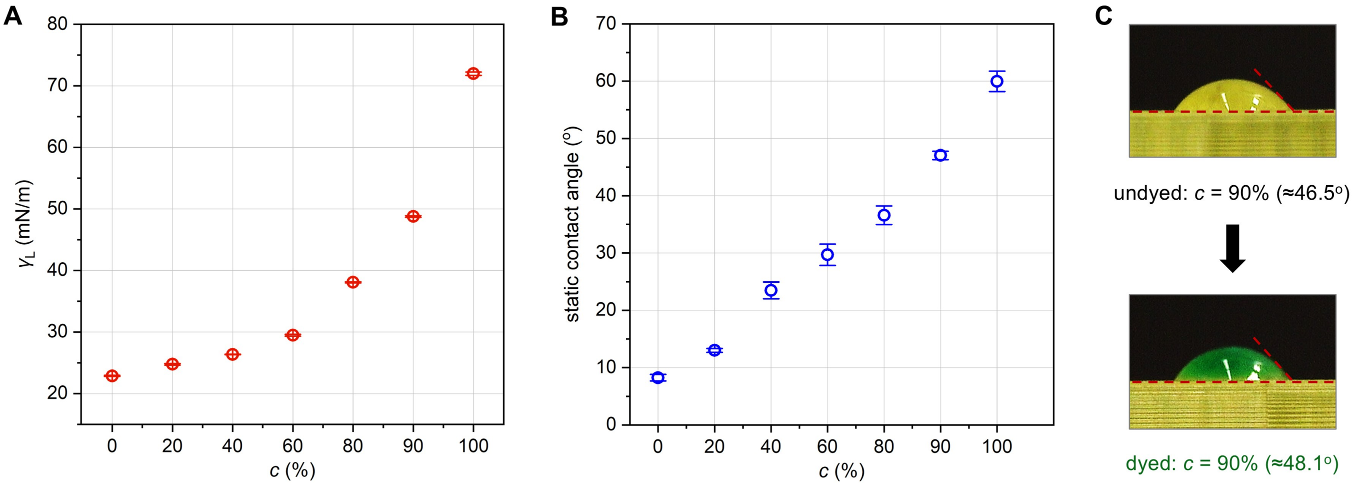

FIG. S3. (A) The liquid surface tension under different c. (B) The liquid apparent contact angle under different c. (C) Contact angle comparison of $c = 90\%$ liquid: without dye (≈46.5°); with the methylene blue dye (≈48.1°). Error bars denote the SD.

III. MECHANISM ANALYSIS OF LIQUID MANIPULATION

Figure S4 presents the directional transport behavior of liquids with extremely low/high surface tension on the curvature-ratchet surfaces. For $c = 0\%$ liquid, the extremely low contact angle displayed at the interface leads to a decrease in Laplace pressure resistance in both the positive and negative x-directions, allowing the liquid to exhibit bidirectional spreading (Figure S4A-i). When the surface is changed to $\beta = 120^{\text{o}}$, the extra tilt feature can correct the bidirectional spreading of $c = 0\%$ liquid, transforming it into directional transport along the negative x-direction (Figure S4A-ii).

We then consider the spreading of 20 M/L potassium hydroxide (KOH) solution with an extremely high surface tension (98 mN/m). On $\beta = 90^{\text{o}}$ surface, symmetric top structure leads to the same advancing contact angles ($\theta_{x\text{-}}$ and θ_{x+}), thus the curvature feature still dominates the KOH solution to exhibit mode-1 spreading toward the negative x-direction (Figure S4B-i). However, when the liquid spreads on $\beta = 120^{\text{o}}$ surface, the inherent advancing contact angle θ_{a0}, under asymmetric top structure, presents a smaller apparent contact angle in the positive x-direction ($\theta_{x+} < \theta_{x\text{-}}$), indicating less resistance in the positive x-direction. This causes the reentrant liquid to transport toward the direction against the tilted direction of ratchets (Figure S4B-ii), demonstrating a reentrant transport mode reported in some previous works [1, 2].

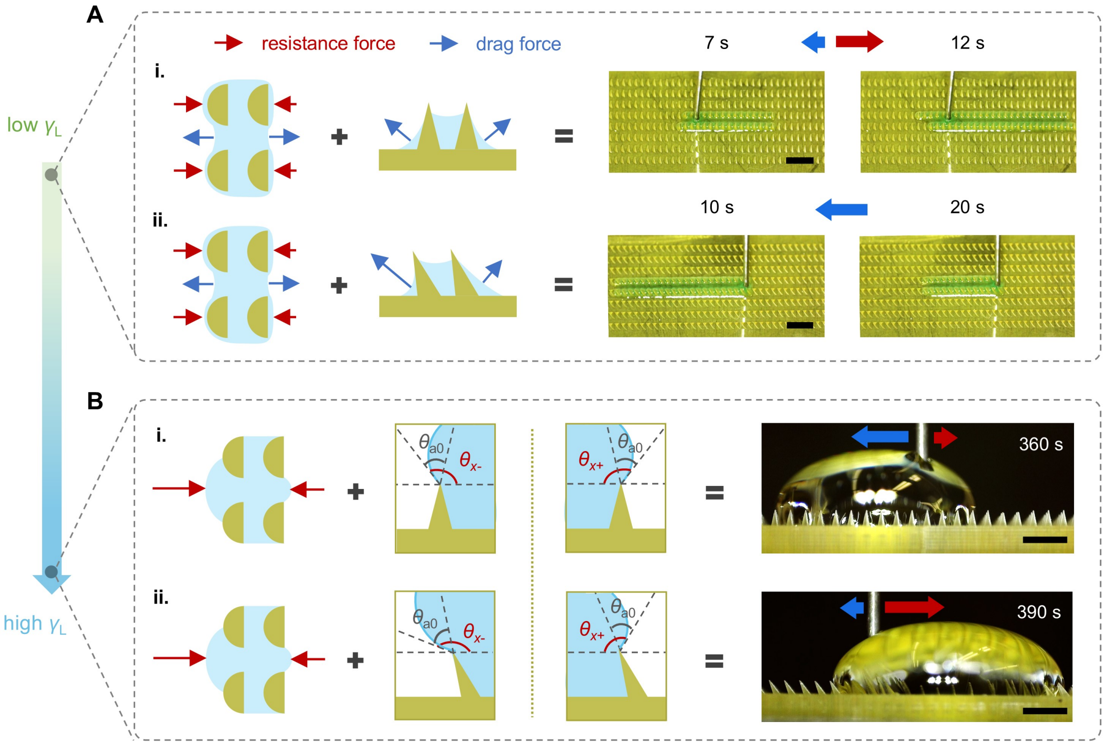

FIG. S4. (A) The spreading of $c = 0\%$ liquid on i) $\beta = 90^{\text{o}}$ surface (7 s and 12 s); ii) $\beta = 120^{\text{o}}$ surface (10 s and 20 s). (B) The spreading of 20 M/L KOH solution on i) $\beta = 90^{\text{o}}$ surface (360 s); ii) $\beta = 120^{\text{o}}$ surface (390 s). Scale bars: 3 mm (A), and 2 mm (B)

In Figure S5, we assess the impact of different fluid regimes on liquid spreading, primarily considering whether inertial effects can be neglected. When injecting liquids with a flow rate of 100 µL/min across a concentration range of $c = 0$–100%. The Weber number (We), calculated according to Equation 3 (in the main text), remains around 0.001 (Figure S5A), indicating the ignorable inertial effect. As inertial effects increase (i.e., with higher injection flow rates), the liquid may break the pinning on both sides, transitioning from directional to bidirectional spreading. We examine scenarios with We of 1 and 5, which represent regimes where inertial forces and interfacial forces are comparable, and where inertial forces dominate, respectively. Figure S5B illustrates the required injection flow rates for different liquids in these two scenarios. With progressively higher initial injection flow rates, increasing cases of directional transport in the original phase diagram (Figure 2G of the main text) become uncontrollable bidirectional transport cases (Figures S5C and Figures S5D). This emphasizes the importance of selecting the appropriate fluid regime for effective directional liquid transport in practical applications.

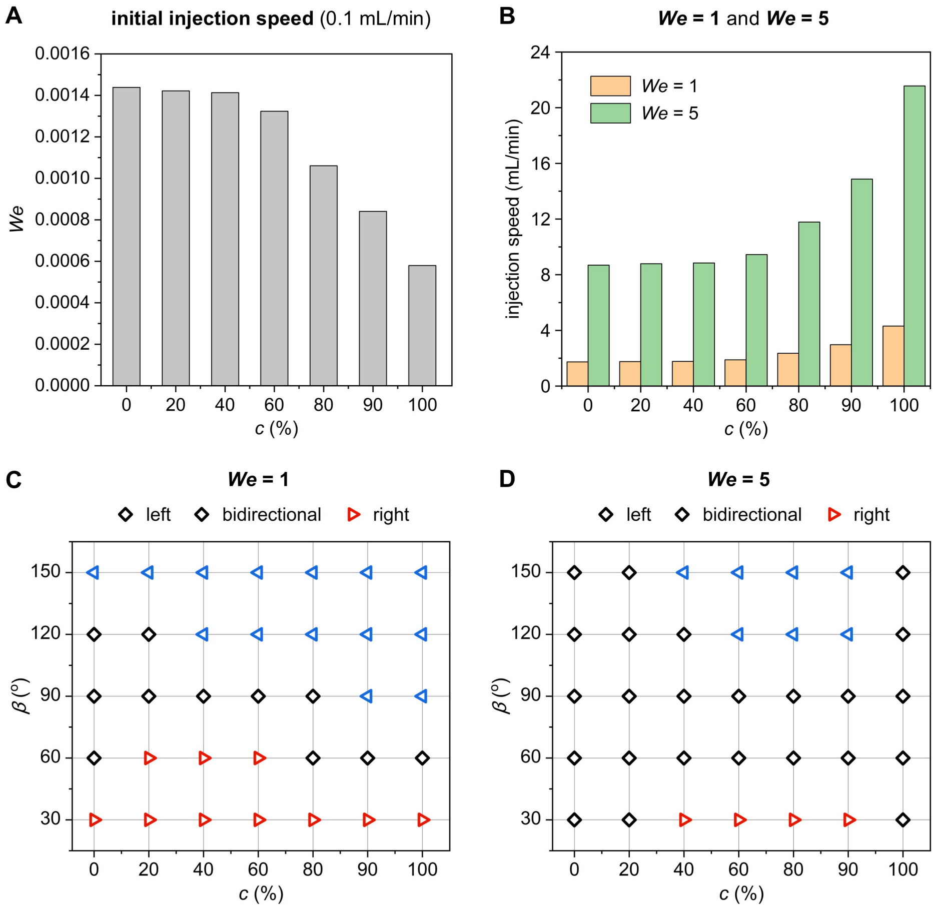

FIG. S5. (A) We of different liquids injected at a flow rate of 100 µL/min. (B) The injection flow rate of different liquids to achieve $We = 1$ and 5. (C) The updated phase diagram at $We = 1$. (D) The updated phase diagram at $We = 5$.

We demonstrate the cross-scale applicability of curvature-ratchets by scaling down the ratchet by a factor of 4 and scaling it up by a factor of 3 (Figure S6). Like in the original scale, the directional transport behavior of liquid (e.g., $c = 100\%$) on different surfaces still follows the rules shown in Figure 2 of the main text.

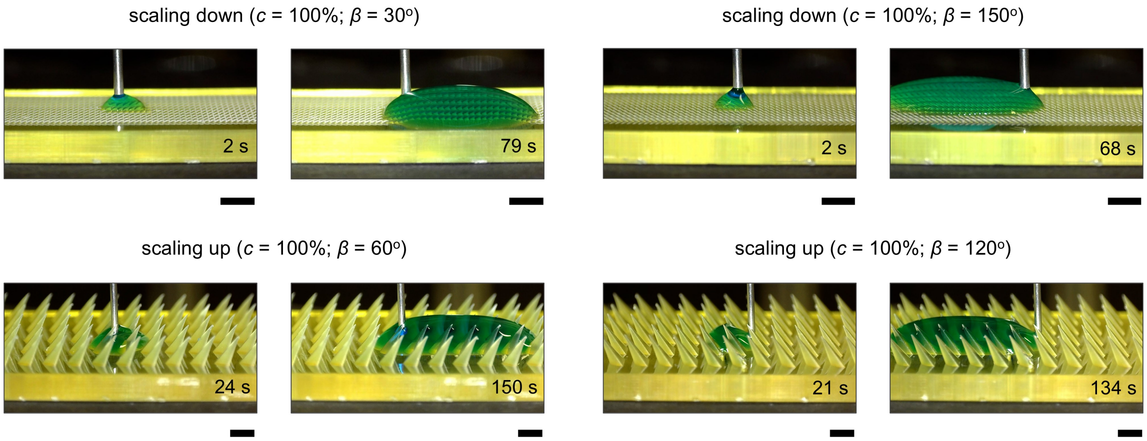

FIG. S6. Evaluation experiments of the cross-scale applicability of curvature-ratchet surfaces. Scale bars: 2 mm.

In Figure S7, we list the size parameters of surfaces with original, reduced, and increased dimensions, whose minimum sizes range from a few microns to hundreds of microns. Their underlying mechanism for directional liquid manipulation relies on asymmetric liquid-air interfaces introduced by the curvature and tilt features, exhibiting cross-scale adaptability for structured surface features at the nanoscale [3], microscale [4], and even millimeter-scale [5]. To balance precise liquid manipulation with processing accuracy and cost-efficiency, structured surfaces at the microscale are often preferred by researchers. Their sizes avoid being too small (nanoscale), which makes complex 3D surface features difficult to fabricate, and too large, which would hinder precise small-scale liquid manipulation.

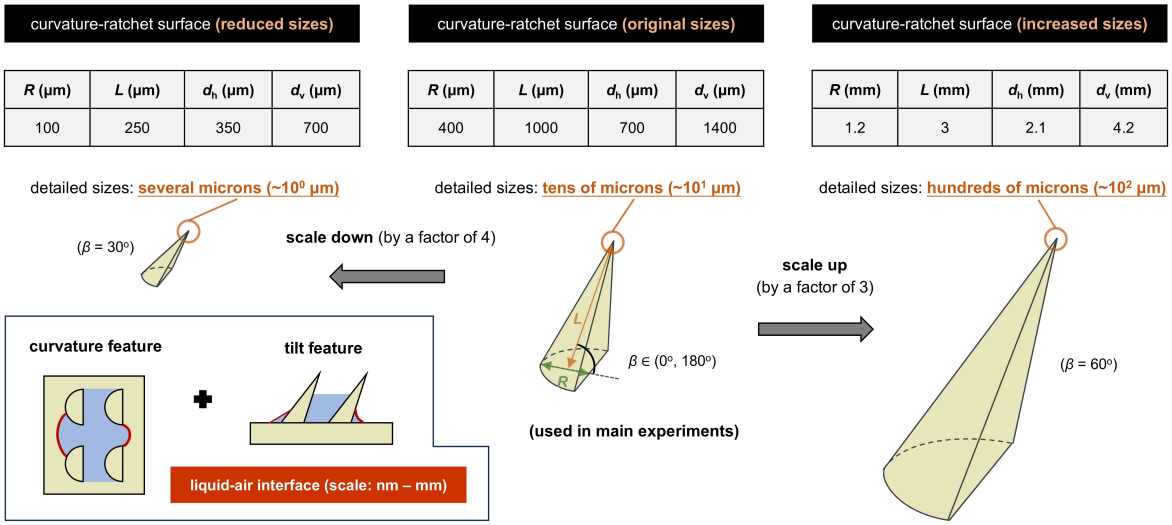

FIG. S7. Cross-scale liquid-air interfaces and selection of appropriate surface feature scales.

In this study, complex directional liquid dynamics on structured surfaces is supported by the curvature-ratchet model. As shown in Figure S8, neither a single curvature feature (where mode-2 directional liquid manipulation is limited by inertial effects) nor a single tilt feature (where liquid behavior primarily responds to inclined structures rather than its surface tension) can simultaneously meet the demands for stable, diverse, and adjustable liquid manipulation involving complex liquid-solid interfacial energy relationships. This is achieved in the curvature-ratchet model (as shown in Figures 2–4 of the main text), which incorporates both fixed curvature and varying tilt features. Meanwhile, this highlights that the design of structured surfaces with multiple asymmetric features is fundamental to exploring sophisticated and smart liquid manipulation capabilities, aligning with the recently proposed design strategies for multimodal liquid manipulation surfaces [1, 6].

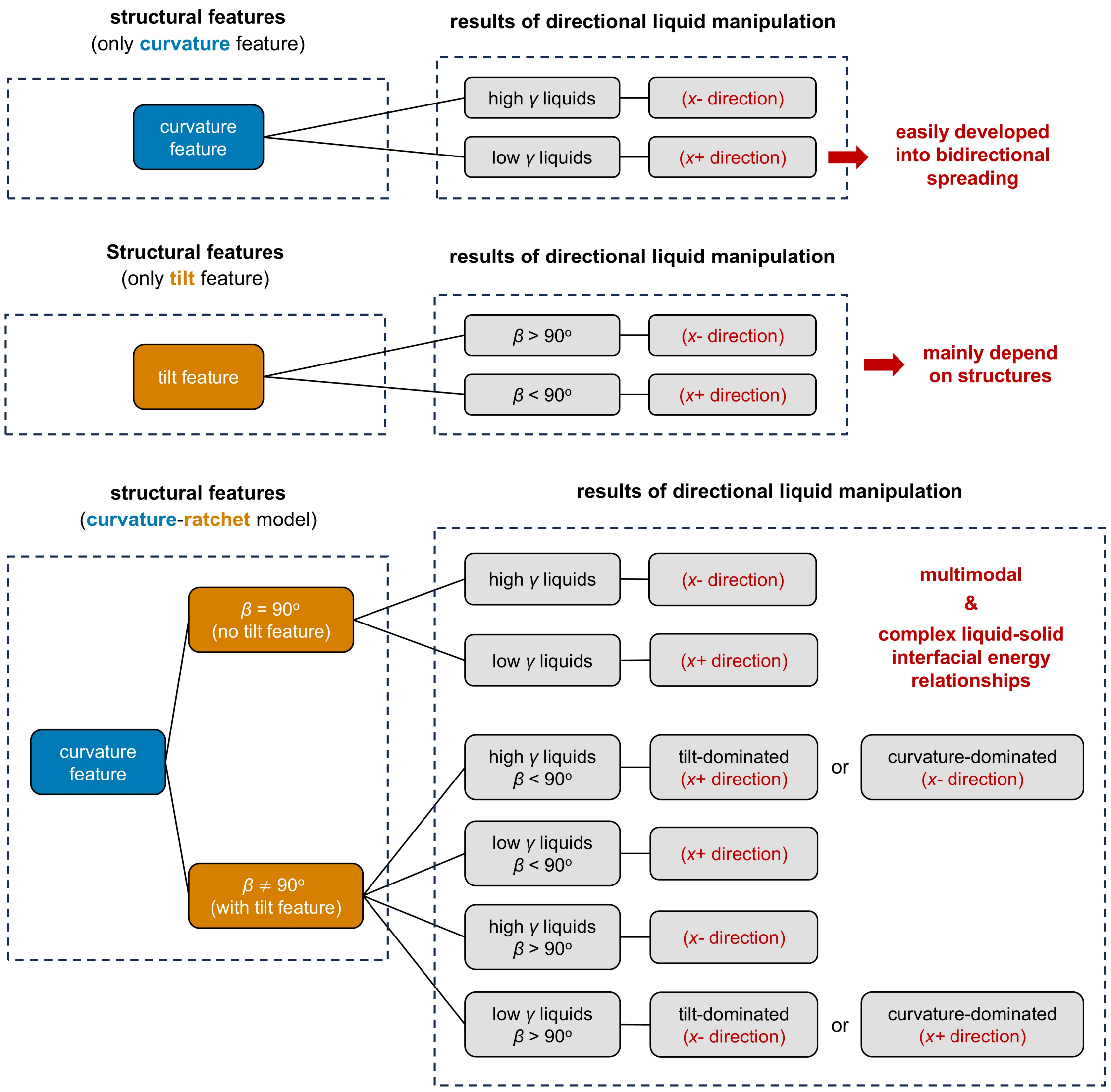

FIG. S8. Comparison of different structured surfaces and their corresponding liquid manipulation results.

IV. THREE LIQUID CONTROL REGIMES

Figure S9 presents the specific data of Figure 3H in the main text (i.e., sample information for the used surfaces and liquids). For untreated, hydrophilic-treated, and Ecoflex surfaces, we selected the ethanol-water mixtures involved in the main experiments: $c = 0\%$ (ethanol), $c = 20\%$, $c = 40\%$, $c = 60\%$, $c = 80\%$, $c = 90\%$ and $c = 100\%$ (DI water). For the hydrophobic-treated surface, to avoid the additional effects of ethanol on the hydrophobic nanoparticles that could compromise experimental accuracy, we only used DI water and utilized three other liquids (with a sufficient surface tension range from 21 mN/m to 62.7 mN/m) to investigate different regimes. Specific sample information, including surface free energy (γ_S), liquid surface tension (γ_L), and ζ, is provided in Figure S9, with each marked by its corresponding regime.

Hydrophobic-treated surface	Liquid 1: silicone oil	Liquid 2: ethylene glycol	Liquid 3: glycerol	Liquid 4: DI water
γ_S = 1.1 mJ/m^2	γ_L = 21.0 mN/m	γ_L = 48.1 mN/m	γ_L = 62.7 mN/m	γ_L = 72.0 mN/m
ζ value	ζ = 0.052	ζ = 0.023	ζ = 0.018	ζ = 0.015

excessive regime

balanced regime

insufficient regime

Untreated surface	Liquid 1: ethanol	Liquid 2: *c* = 20%	Liquid 3: *c* = 40%	Liquid 4: *c* = 60%	Liquid 5: *c* = 80%	Liquid 6: *c* = 90%	Liquid 7: DI water
γ_S = 11.5 mJ/m^2	γ_L = 22.9 mN/m	γ_L = 24.8 mN/m	γ_L = 26.4 mN/m	γ_L = 29.5 mN/m	γ_L = 38.1 mN/m	γ_L = 48.8 mN/m	γ_L = 72.0 mN/m
ζ value	ζ = 0.502	ζ = 0.464	ζ = 0.436	ζ = 0.390	ζ = 0.302	ζ = 0.236	ζ = 0.160

Ecoflex surface	Liquid 1: ethanol	Liquid 2: *c* = 20%	Liquid 3: *c* = 40%	Liquid 4: *c* = 60%	Liquid 5: *c* = 80%	Liquid 6: *c* = 90%	Liquid 7: DI water
γ_S = 46.0 mJ/m^2	γ_L = 22.9 mN/m	γ_L = 24.8 mN/m	γ_L = 26.4 mN/m	γ_L = 29.5 mN/m	γ_L = 38.1 mN/m	γ_L = 48.8 mN/m	γ_L = 72.0 mN/m
ζ value	ζ = 2.009	ζ = 1.855	ζ = 1.742	ζ = 1.560	ζ = 1.207	ζ = 0.943	ζ = 0.639

Hydrophilic-treated surface	Liquid 1: ethanol	Liquid 2: *c* = 20%	Liquid 3: *c* = 40%	Liquid 4: *c* = 60%	Liquid 5: *c* = 80%	Liquid 6: *c* = 90%	Liquid 7: DI water
γ_S = 70.5 mJ/m^2	γ_L = 22.9 mN/m	γ_L = 24.8 mN/m	γ_L = 26.4 mN/m	γ_L = 29.5 mN/m	γ_L = 38.1 mN/m	γ_L = 48.8 mN/m	γ_L = 72.0 mN/m
ζ value	ζ = 3.079	ζ = 2.843	ζ = 2.670	ζ = 2.390	ζ = 1.850	ζ = 1.445	ζ = 0.979

FIG. S9. Sample information of surfaces and liquids in Figure 3H of the main text.

V. DESIGN OF VARIOUS SURFACE ARRAY ARRANGEMENTS

Figures S10–S12 present details of the three types of surface array arrangements. Inspired by the scale lattice arrangement of the dorsal wing surface [7], we design a dislocation surface array arrangement consisting of 31 × 37 ratchets (Figure S10A). Figure S10B displays its SEM images from both front and top views. The combined effect of tilt and curvature enables both high and low surface tension liquids to exhibit a similar fan-shaped spreading in Figure 4A–C of the main text.

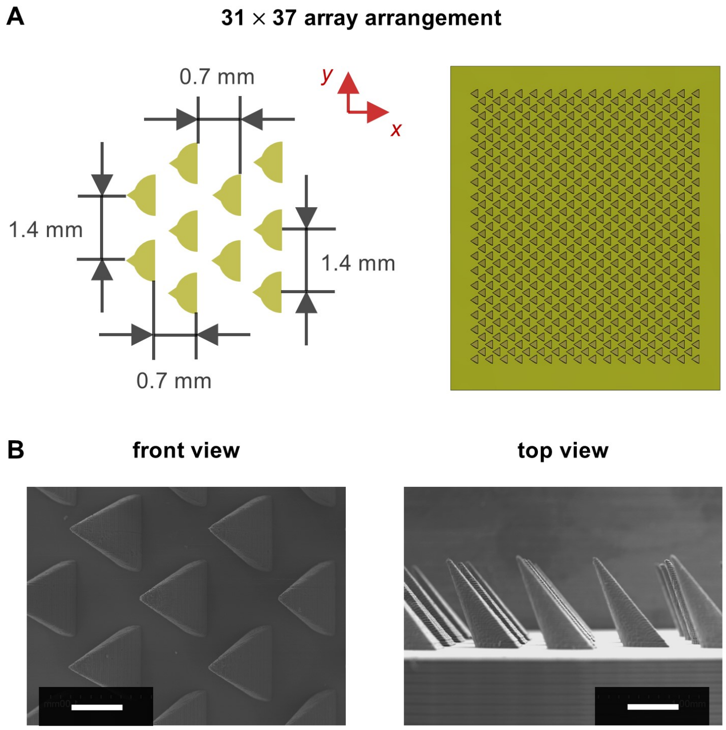

FIG. S10. (A) Illustration of the surface with dislocation array arrangement. (B) SEM images of the surface with dislocation array arrangement. Scale bars: 500 μm.

Inspired by the different-density structure of Ligia exotica [8], we design a gradient spacing surface array arrangement with 28 × 8 ratchets (Figure S11A). Figure S11B displays the combined multiple SEM images of this surface from a top view to show the difference in horizontal spacing d_h. The liquid with $c = 60\%$ originally presents bidirectional spreading. When it encounters a progressively denser array of ratchets, the additional directionality from denser structures makes the liquid spread toward the positive x-direction. However, the injected $c = 90\%$ liquid is not transported to the area with gradient spacing arrangement, thus maintaining its original directionality (Figure S11C and Figure 4E of the main text). In addition, due to the sparse density of the ratchets in the negative x-direction of the left-half surface, the liquid preferentially expands along the y-axis (Figure S11C and Figure 4E of the main text).

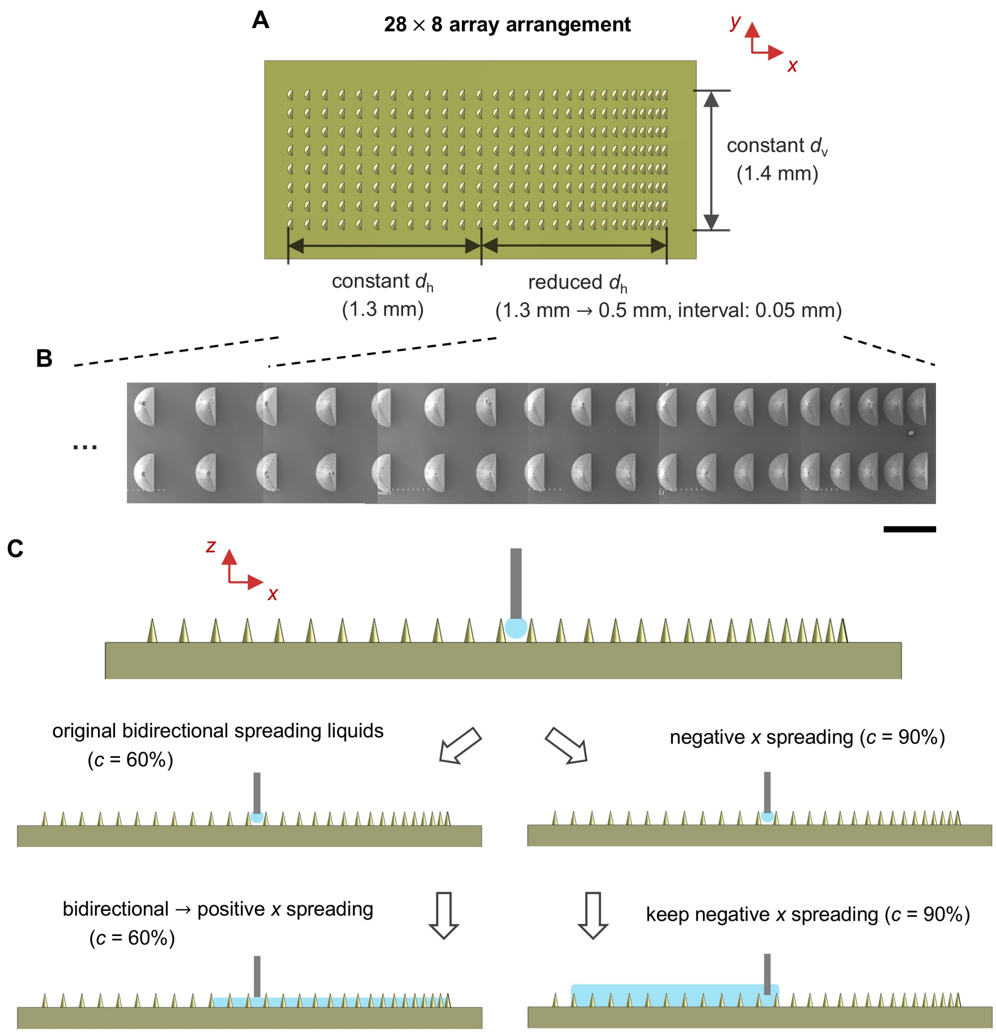

FIG. S11. (A) Illustration of the surface with gradient spacing array arrangement. (B) SEM images of the surface with gradient spacing array arrangement. (C) The directional transport of $c = 60\%$ and $c = 90\%$ liquids. Scale bar: 1 mm.

Inspired by the circumferential spine arrangement of the cactus (Figure S12A), we provide a varied titled angle design for the surface array arrangement (with 40 $\times$ 8 ratchets) in Figure S12B. Figure S12C presents the combined multiple SEM images of this surface from a front view to show the varying structural angle β. This arrangement allows the liquid (e.g., c = 80%) to present back-and-forth transport (states ①–③) when meeting different pinning positions (Figure S12D and Figure 4H of the main text). As for c = 100% liquid, the increased Laplace pressure from the bottom curvature in the positive x-direction hinders the liquid from overcoming the pinning between the ratchets' gaps (state ①), and instead promotes the accumulation of liquid across ratchets (state ②), exhibiting a reentrant transport like the result in Figure S4B-ii. Therefore, c = 100% liquid will be continuously directed to the negative x-direction, rather than presenting back-and-forth transport.

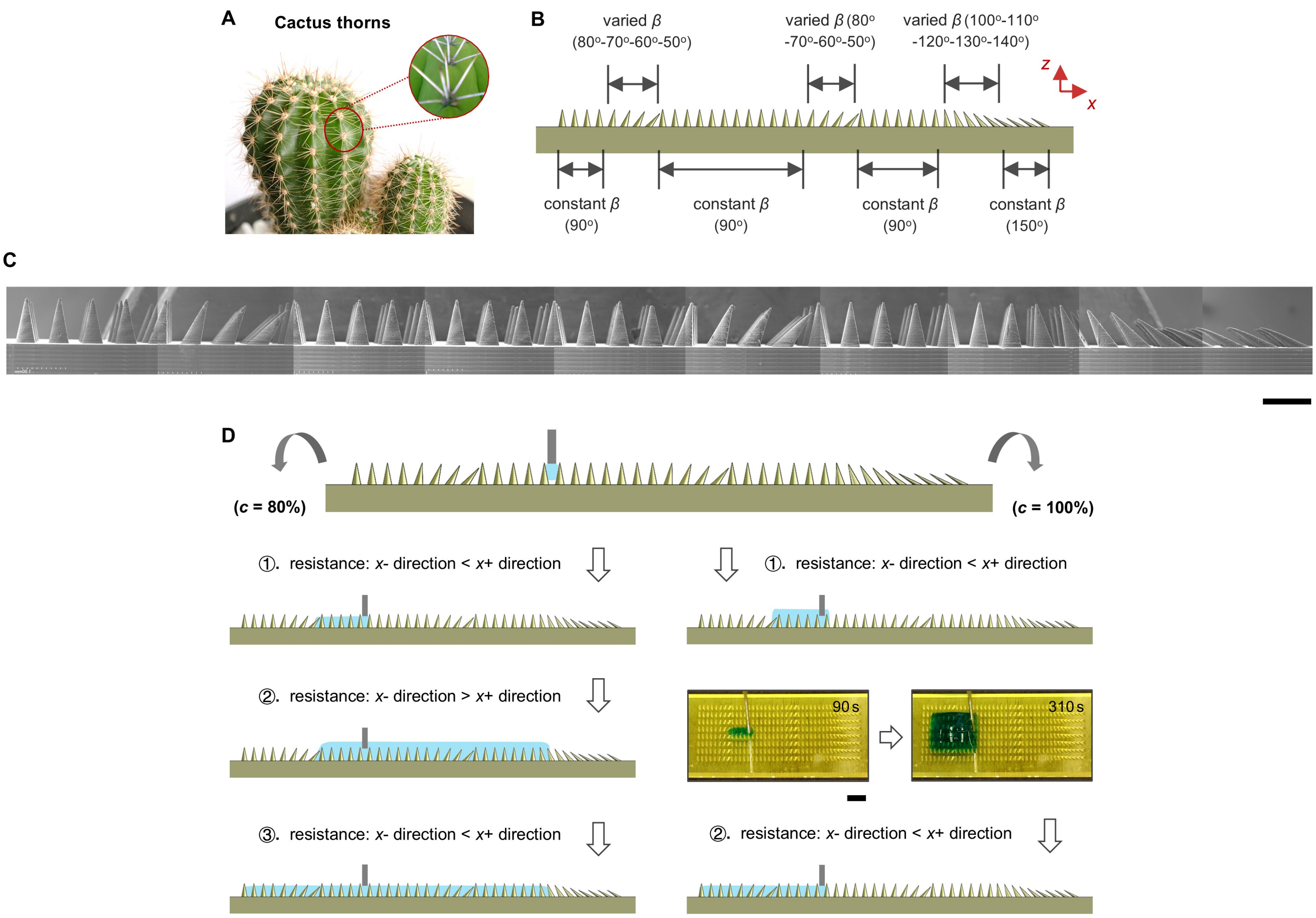

FIG. S12. (A) Illustration of the circumferential spine arrangement of the cactus. (B) Illustration of the array arrangement with varied titled angles. (C) SEM images of the surface with varying β array arrangement. (D) The back-and-forth transport of c = 80% liquid and directional spreading of c = 100% liquid. Scale bars: 1 mm (C); 3 mm (D).

VI. LIQUID-BASED INFORMATION ENCRYPTION TECHNIQUE

Figure S13 shows experiments of the three information acquisition scenarios (liquid injection speed: 50 µL/min), including no information (low ζ), right information (suitable ζ), and wrong information (high ζ). $c = 100\%$ liquid dyed with safranin falls off the surface, presenting no information (at 32 s) after injection, showing no information; $c = 50\%$ liquid dyed with methylene blue shows the right information, i.e., number 2 (at 47 s); $c = 0\%$ liquid dyed with crystal violet breaks the surface pinning arbitrarily during the whole spreading process, showing the wrong information, i.e., number 8 (at 42 s).

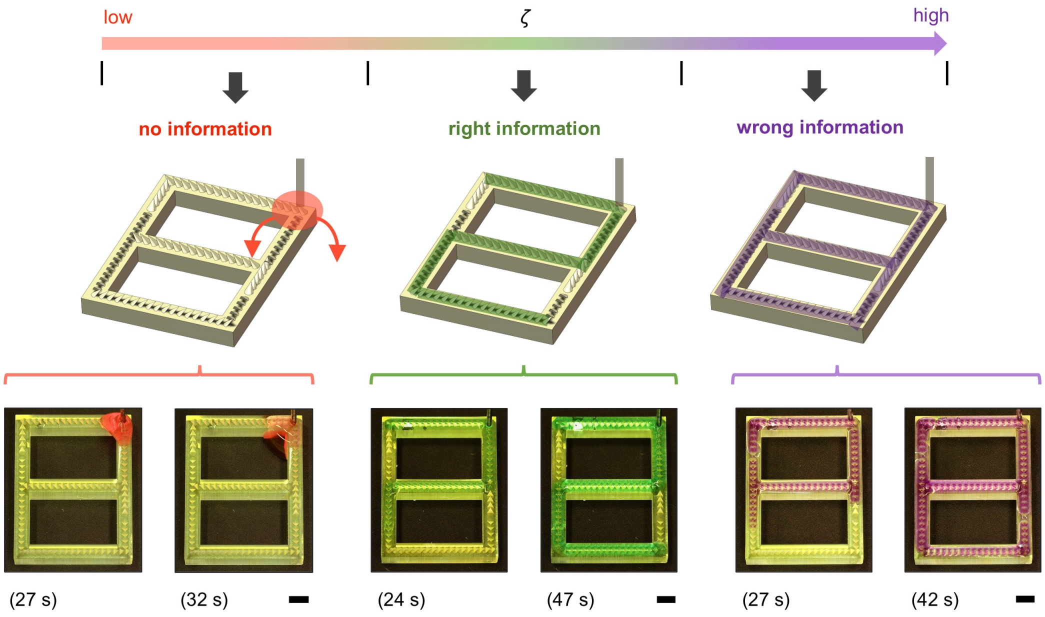

FIG. S13. Experiments of the three information acquisition scenarios in the liquid-based information encryption technique. Scale bars: 2 mm.

Figure S14 presents the array arrangements for the surfaces encoded ten-digit information. The pinning design at key turning positions ensures the correct information display. Under these designs, c = 50% liquid injected at 50 μL/min displays the numbers 0–9 at 10 s, 46 s, 29 s, 20 s, 32 s, 35 s, 14 s, 31 s, 30 s, and 27 s.

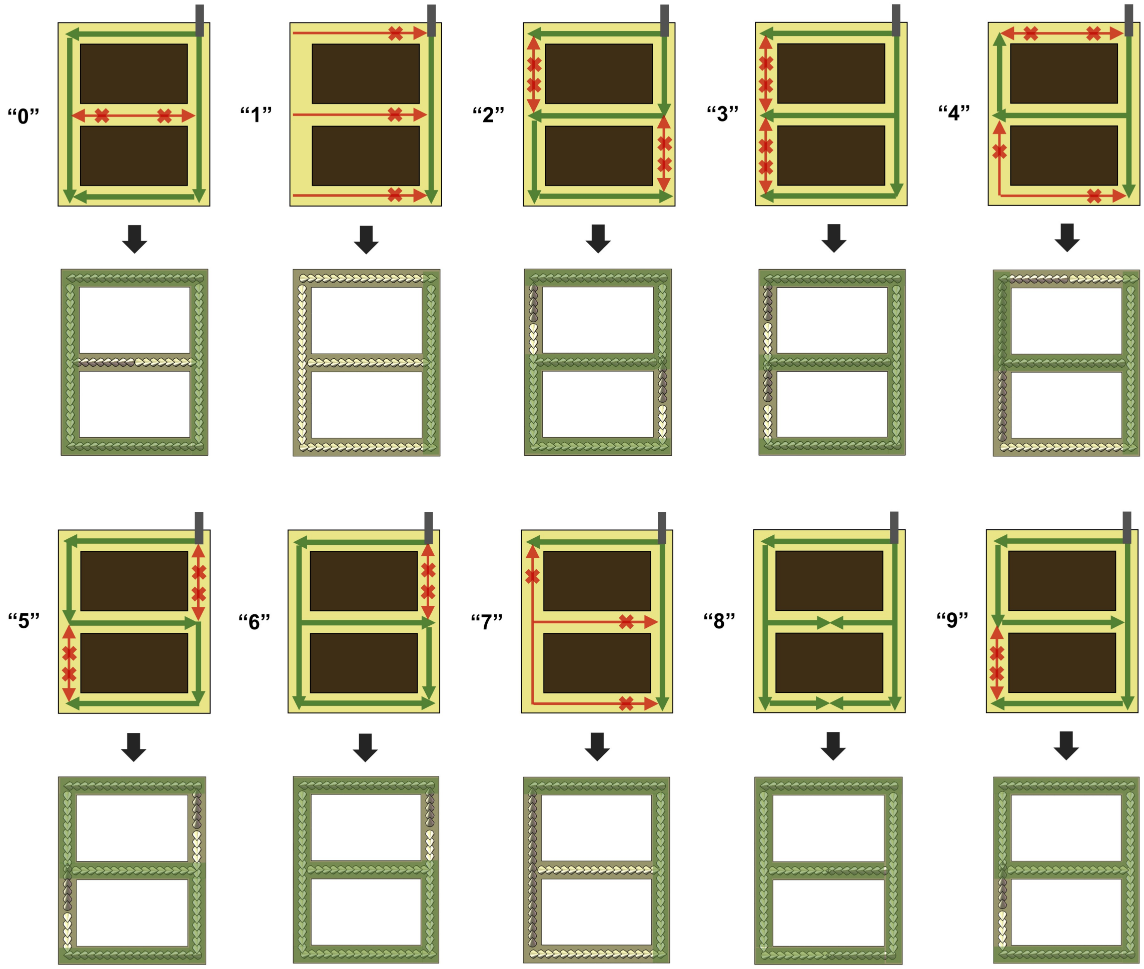

FIG. S14. The array arrangements for the surfaces encoded ten-digit information.

Legends for Movies S1-S4

Movie S1. Directional liquid manipulation on curvature-ratchet surfaces

Movie S2. Liquid spreading on untreated, and hydrophilic/hydrophobic-treated surfaces

Movie S3. Multiple liquid manipulation patterns on different surface array arrangements

Movie S4. Liquid-based information encryption technique

[1] S. Feng, P. Zhu, H. Zheng, H. Zhan, C. Chen, J. Li, L. Wang, X. Yao, Y. Liu, and Z. Wang, Three-dimensional capillary ratchet-induced liquid directional steering, Science **373**, 1344 (2021).

[2] J. Miao and A. C. Tsang, Reconfigurability-encoded hierarchical rectifiers for versatile 3d liquid manipulation, Adv. Sci. **11**, 2405641 (2024).

[3] K.-H. Chu, R. Xiao, and E. N. Wang, Uni-directional liquid spreading on asymmetric nanostructured surfaces, Nat. Mater. **9**, 413 (2010).

[4] H. Chen, P. Zhang, L. Zhang, H. Liu, Y. Jiang, D. Zhang, Z. Han, and L. Jiang, Continuous directional water transport on the peristome surface of nepenthes alata, Nature **532**, 85 (2016).

[5] H. Geng, H. Bai, Y. Fan, S. Wang, T. Ba, C. Yu, M. Cao, and L. Jiang, Unidirectional water delivery on a superhydrophilic surface with two-dimensional asymmetrical wettability barriers, Mater. Horiz. **5**, 303 (2018).

[6] S. Sun, Y. Zhang, S. Wu, and L. Wang, In situ multi-directional liquid manipulation enabled by 3d asymmetric fang-structured surface, Adv. Mater. **36**, 2407034 (2024).

[7] B. D. Wilts, A. Matsushita, K. Arikawa, and D. G. Stavenga, Spectrally tuned structural and pigmentary coloration of birdwing butterfly wing scales, J. R. Soc. Interface **12**, 20150717 (2015).

[8] G. Liu, L. Zhang, Y. Gan, Y. Wang, D. Chen, Y. Dai, L. Feng, P. Zhang, and H. Chen, Liquid transport with direction guidance and speed enhancement from gradient and magnetized micro-cilia surface, Appl. Phys. Lett. **120** (2022).
